# Case report of epithelioid sarcoma of the vulva

**DOI:** 10.3389/fmed.2026.1896749

**Published:** 2026-08-07

**Authors:** Fang Chen, Feng Bai, Wenjuan Li

**Affiliations:** Department of Gynecology, People’s Hospital of Liaoning, Shenyang, China

**Keywords:** epithelioid sarcoma, female urogenital diseases, follow-up, rare tumor, vulvar neoplasms

## Abstract

**Background:**

Epithelioid sarcoma is an extremely rare malignant mesenchymal tumor, accounting for less than 1% of all types of sarcomas. When it occurs in the vulva, it is even less common, exhibiting high invasiveness and a significant risk of recurrence. Epithelioid sarcoma that develops in the deep layers of the vulva is often insidious and may clinically present as a vulvar abscess, which can easily lead to misdiagnosis as a benign lesion.

**Case:**

A 56-year-old female patient presented with a rapidly growing, painful mass measuring 3 cm in diameter on the left vulva. She visited multiple hospitals over the course of 3 months and received anti-inflammatory treatment as well as incision and drainage for a local abscess, but showed no improvement. The patient eventually sought care at our hospital due to progressive ulceration of the vulva, increasing size of the lesion, and swelling of the buttock skin accompanied by septic shock. After stabilizing her general condition, an excision of the vulvar lesion and a fasciotomy for decompression and drainage of the buttock were performed. Postoperative pathology confirmed a diagnosis of vulvar epithelioid sarcoma (proximal type). The patient subsequently died from septic shock associated with multiple organ failure.

**Conclusion:**

From a clinical perspective, vulvar epithelioid sarcoma can present similarly to various benign vulvar lesions, such as Bartholin gland abscesses and infectious granulomas, leading to potential misdiagnosis. Here, we share our experience in the diagnosis and management of a patient with vulvar epithelioid sarcoma to provide a cautionary perspective for the clinical care of comparable cases.

## 1 Introduction

Epithelioid sarcoma (ES) is a rare malignant mesenchymal tumor characterized by epithelioid cell morphology and is classified into proximal-type (proximal-type epithelioid sarcoma, PES) and distal-type (distal-type sarcoma, DES). The classic type (distal type) typically occurs in the extremities and exhibits a pseudogranulomatous growth pattern, while the proximal type (large cell type) is more commonly found in the trunk. Reports in the literature indicate that the distal type is approximately twice as prevalent as the proximal type, with proximal epithelioid sarcoma commonly affecting individuals aged 20 to 65 years (1). It frequently arises in the deep soft tissues of the vulva or perineum, making it an extremely rare and diagnostically challenging disease that is prone to misdiagnosis. Clinically, proximal epithelioid sarcoma presents as a rapidly growing superficial mass. Approximately 50 cases of proximal-type epithelioid sarcoma (PES) have been reported globally, with most patients undergoing local excision as initial treatment (1). Here, we report a case of proximal-type epithelioid sarcoma of the vulva in a 56-year-old female patient. The patient required care at multiple hospitals over the course of her illness due to disease progression, experienced persistent diagnostic delays throughout her entire care pathway, which prevented the timely initiation of therapeutic intervention, and ultimately succumbed to the disease (Table 1). Through this case, we aim to alert clinicians to the clinicopathological features of this malignancy, and underscore the importance of early diagnosis and prompt treatment for this condition.

**TABLE 1:** Timeline of clinical presentation, investigations, and management for the index case.

Timeline	Clinical manifestations and examination findings	Medical management
Jun 2022	56-year-old female patient presented to a local hospital with a painful ∼3 cm mass on the left vulva, initially diagnosed as a Bartholin gland abscess	Received anti-inflammatory therapy with no symptomatic improvement
Aug 2022	The mass showed significant enlargement and intensified pain	Underwent incision and drainage of the abscess; received postoperative anti-inflammatory therapy
Aug–early Sep 2022	Persistent non-healing of the surgical incision	Visited two additional hospitals for wound care and anti-inflammatory management, with no alleviation of symptoms
Sep 5, 2022 +2 weeks	Surface ulcer (0.5 cm at onset) expanded to ∼6 cm over 2 weeks with hemorrhage; concurrent left buttock erythema and edema observed	No specific intervention
Sep 9–15, 2022	Developed altered mental status; laboratory tests revealed hemoglobin of 61 g/L and postprandial blood glucose of 22.4 mmol/L	Transferred to our hospital for admission and further evaluation

## 2 Materials and methods

### 2.1 Clinical data

Physical Examination: The vulva is normally developed, with significant swelling of the left labium majus, which appears slightly brownish. The lower third is ulcerated, measuring 6 × 4 × 3 cm, necrotic wound bed with purulent exudate. There is marked tenderness upon palpation. Additionally, the skin over the left buttock is swollen, extending to the upper quarter of the thigh, with a hard texture, marked tenderness, and elevated skin temperature.

Past Medical History: The patient has a 2-year history of diabetes mellitus, with irregular blood glucose monitoring and inconsistent medication adherence. Over ten years ago, she underwent an abdominal total hysterectomy with bilateral adnexectomy due to uterine fibroids, with postoperative pathology confirming a benign diagnosis. She denies any other medical history or family history of disease.

### 2.2 Treatment methods

**FIGURE 1:**
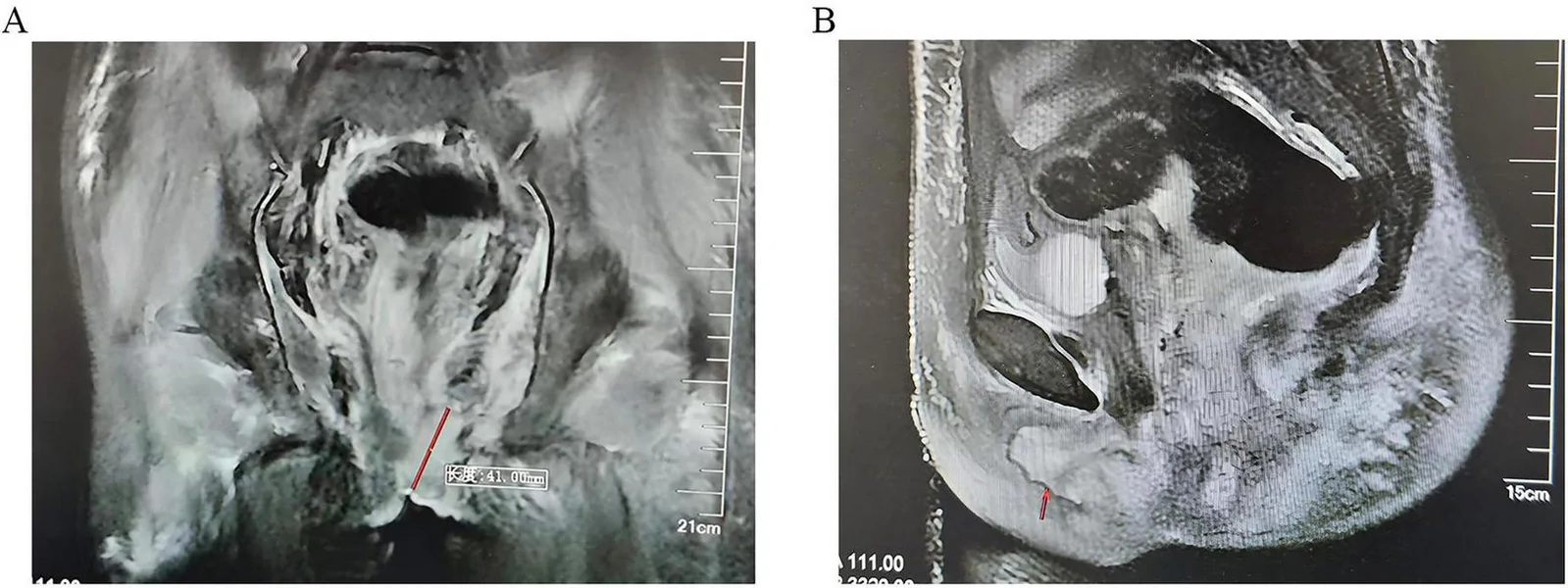
Enhanced magnetic resonance imaging of the vulva. **(A)** Coronal view (with red markings indicating the size of the lesion). **(B)** Sagittal view (with red arrows indicating the location of the lesion).

**FIGURE 2:**
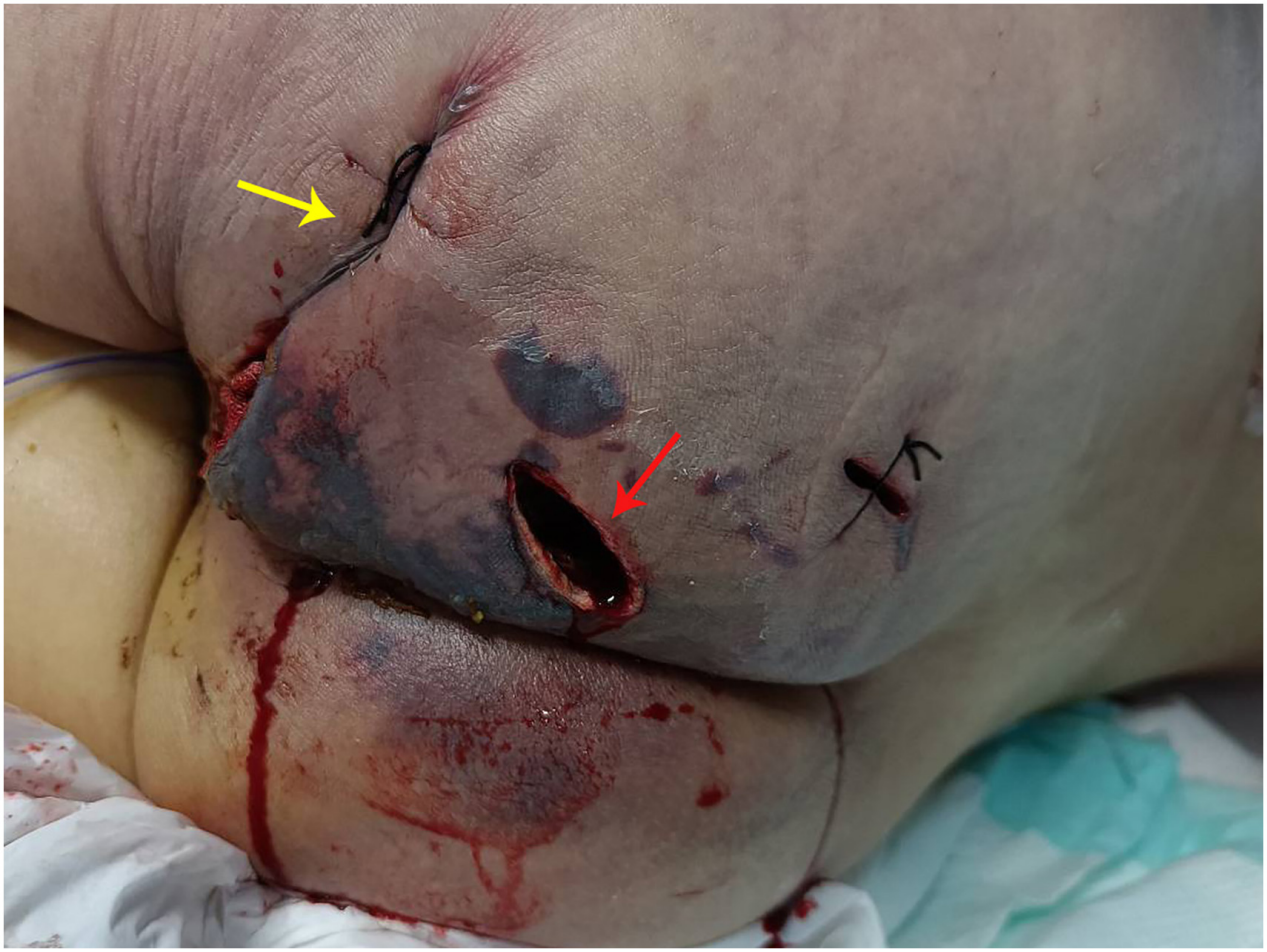
Left gluteal fasciotomy for decompression and drainage (yellow arrow) and buttock fistulotomy (red arrow).

**FIGURE 3:**
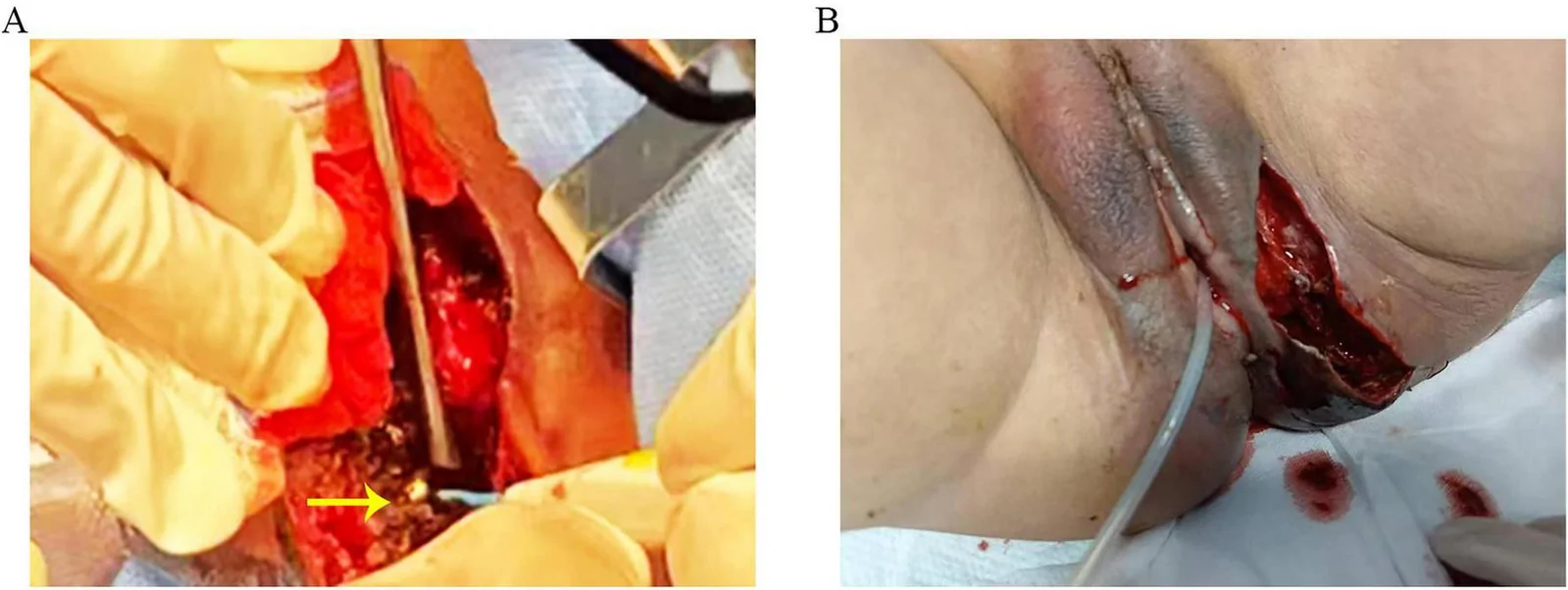
Intraoperative findings and postoperative photographs of the vulva. **(A)** Intraoperative view showing a hidden mass beneath the vulvar infection site (yellow arrow). **(B)** Postoperative photograph of the vulva.

Upon admission, the patient was considered to have vulvar infection complicated by diabetes, leading to severe infection and septic shock due to recurrent non-healing wounds. She received anti-infective treatment, shock management, and blood glucose regulation. Enhanced magnetic resonance imaging of the vulva revealed infection in the perineum and pelvic floor, with swelling and exudation of the pelvic floor muscles, buttocks, and soft tissues of the left thigh (Figures 1A, B). On post-admission day 2, the patient’s septic shock worsened. Following consultation with the orthopedic surgery team, the working clinical impression was that persistent non-healing of the vulvar incision and drainage (I&D) site, in the context of impaired local tissue perfusion, had driven secondary infection and progression to necrotizing fasciitis. The patient subsequently underwent left gluteal fasciotomy with decompressive drainage (Figure 2). All serial sputum, purulent exudate, and blood cultures returned negative results. Despite this, septic shock persisted with inadequate control, and the patient developed gluteal muscle necrosis and uncontrollable bleeding from the perineum, further exacerbating her septic shock. After multidisciplinary consultation, she underwent surgery under general anesthesia. During the procedure, a hidden mass was observed beneath the localized infection site in the vulva, and the affected tissue was excised (Figures 3A, B). Additionally, a fistulotomy was performed on the buttock (Figure 2). Postoperatively, she was transferred to the ICU for shock management, infection control, intermittent blood transfusions, and plasma support therapy; however, the response was poor. The patient subsequently experienced progressive septic shock accompanied by organ failure, and her family decided to withdraw life support, leading to her death.

## 3 Pathological results

**FIGURE 4:**
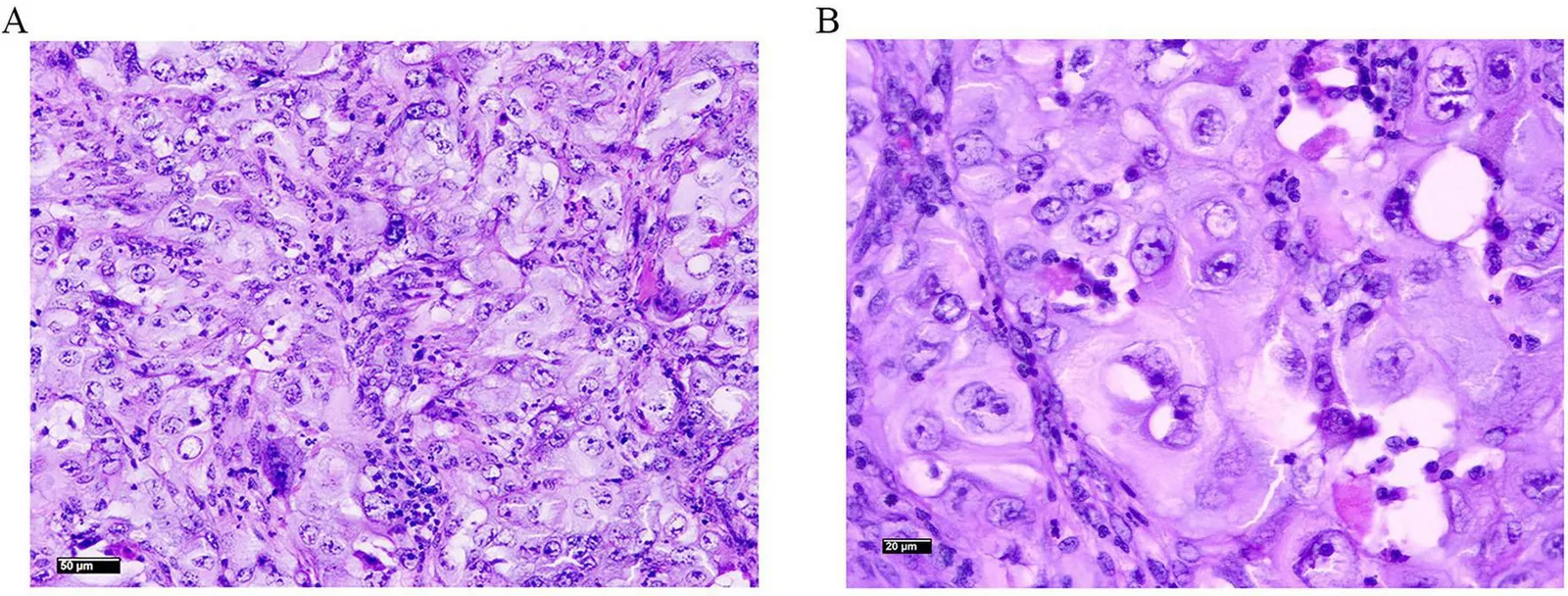
Photomicrographs of the initial resected specimen stained with H&E revealed tumor cells with a rhabdomyoblast-like morphology and prominent nucleoli, with focal areas showing clear cell change. **(A)** 200 × magnification; **(B)** 400 × magnification.

Histopathological Findings: Hematoxylin and eosin (H&E) staining: The tumor tissue showed abundant atypical epithelial-like cells under microscopy, with a diffuse distribution pattern. The tumor cells were large, pleomorphic, with markedly enlarged, hyperchromatic nuclei, and prominent atypia (Figures 4A, B). Immunohistochemistry: CK (+); S-100 (−); INI1 (−, loss of expression); CK7 (scattered +); MyoD1 (−); R (−); Ki-67 (80% +); CK5/6 (focal +); P40 (focal +); P63 (focal +); Vimentin (+); EMA (+); ERG (−); ER (−); CD31 (−); CD34 (+).

Diagnosis: Proximal-type vulvar epithelioid sarcoma(T1b Nx Mx). Nerve invasion: Extensive; Histological grade: Grade III; Tumor necrosis: Positive (+).

## 4 Discussion

Vulvar sarcomas account for less than 5% of all vulvar tumors (2), with smooth muscle tumors being the most common type. Epithelioid sarcoma of the vulva is extremely rare, comprising only about 1% of soft tissue sarcomas. Approximately 50 cases have been reported in the existing English literature, with the disease being most common in patients aged 35 to 50 years. These tumors most frequently occur in the labia majora, followed by the Bartholin gland region, clitoris, and labia minora (3). Clinically, they are often misdiagnosed as benign lesions. In this case, the lesion occurred in the Bartholin gland area, presenting as a painful vulvar abscess. Additionally, the patient had poorly controlled diabetes, which is a risk factor for Bartholin gland abscesses, potentially misleading clinical diagnosis. During surgery under general anesthesia at our hospital, a hidden mass was discovered beneath the abscess, and the deep location of the lesion contributed to the delay in diagnosis. This highlights the importance of thorough clinical examination and differential diagnosis.

In terms of immunohistochemistry, the loss of INI1 protein or SMARCB1 (the gene that encodes INI1) is considered a key diagnostic feature for both proximal-type and distal-type epithelioid sarcoma, and this phenomenon is observed in over 90% of such cases (2, 4). The patient in this case also exhibited loss of INI1 expression, with tumor cells showing positive reactivity for epithelial markers such as AE1/AE3 and EMA. Additionally, there was positive reactivity for CD34 (a mesenchymal marker). The positive result for CD34 aids in the differential diagnosis of tumors that are similar to epithelioid sarcoma. The typical histomorphological features of proximal-type vulvar epithelioid sarcoma include a growth pattern comprising solid nests and perivascular pseudorosettes, epithelioid tumor cells with prominent nucleoli, characteristic geographic (map-like) amorphous necrosis, and marked nuclear pleomorphism (3). This high-grade neoplasm requires differentiation from other easily confused sarcomatous and carcinomatous lesions, including conventional (distal-type) epithelioid sarcoma, synovial sarcoma, malignant peripheral nerve sheath tumor, and sarcomatoid carcinoma (5). Immunohistochemical analysis demonstrates that approximately 95% of PTES cases show complete loss of INI1 protein expression, a pathognomonic diagnostic hallmark of this entity (6); this finding, combined with diffuse positivity for epithelial membrane antigen and vimentin, supports definitive diagnosis. Furthermore, the Ki-67 proliferation index can be used to quantify tumor proliferative activity and assess associated metastatic and recurrence risk (7). Accurate pathological diagnosis constitutes the cornerstone for guiding subsequent individualized treatment planning.

The long-term prognosis for epithelioid sarcoma (ES) is relatively poor, with 5- and 10-year overall survival rates ranging from 45 to 70% and 45 to 66%, respectively. Among the 50 cases published, most patients (approximately 60%) showed no evidence of disease during the follow-up period, with a mean follow-up time of 40 months (standard deviation of 39 months). In contrast, 28% of patients died due to the disease, with a mean follow-up time of 17 months (standard deviation of 20 months) (8). Studies have shown that deeper-seated lesions of epithelioid sarcoma are associated with lower overall survival rates, which partially explains the poorer prognosis of proximal-type epithelioid sarcoma (PES). For localized epithelioid sarcoma (ES), surgical resection remains the main treatment modality. According to relevant studies, neoadjuvant or adjuvant therapy may be recommended, as it is associated with a reduction in recurrence rates (9). In an early study involving 37 patients with ES, all patients underwent initial treatment that consisted of surgery, ranging from local excision to radical vulvectomy, with some patients also undergoing lymphadenectomy or not, primarily depending on the presence of distant metastasis (4, 10). Several studies suggest that the surgical margins for local excision should be at least 2 cm, rather than opting for radical vulvectomy. However, the optimal treatment strategy for ES has yet to be defined. The precise role of adjuvant radiotherapy and/or chemotherapy in the management of ES remains unclear (11). Due to the highly aggressive nature of ES and its poor prognosis, there are currently no established standardized treatment guidelines. Previous studies have shown that even patients with metastatic ES who receive palliative chemotherapy still have a poor prognosis.

Epithelioid sarcoma of the proximal type is characterized by near-universal loss of SMARCB1/INI1, resulting in dysfunction of the SWI/SNF chromatin-remodeling complex (12). This molecular alteration confers resistance to conventional chemo- and radiotherapy and is associated with a poor prognosis in advanced disease. Targeted therapy constitutes the central systemic approach for PTES. mTOR inhibitors achieve objective response rates (ORR) of 10–20 %, disease control rates (DCR) of 50–70 %, and median progression-free survival (PFS) of 4–6 months (13). Anti-angiogenic tyrosine-kinase inhibitors yield ORR of 15–25 % and median PFS of 3–5 months, often stabilizing disease and enabling R0 resection in some initially unresectable cases (14). Overall, targeted strategies extend median overall survival from historical controls of 6–12 months to 18–24 months, providing a paradigm for molecularly driven diagnosis and treatment of rare sarcomas. However, the evidence base derives mainly from small retrospective series; prospective validation is lacking, and aside from INI1 loss, no robust predictive biomarkers have been established. Future efforts should focus on prospective studies anchored to PTES molecular phenotype and investigate combination targeted regimens to improve patient outcomes.

In summary, proximal-type vulvar epithelioid sarcoma is an extremely rare and highly aggressive tumor that typically occurs in middle-aged women. The diagnosis of this disease relies on biopsy examination and the absence of INI1 immunohistochemical expression. For localized lesions, surgical treatment remains the preferred approach, while the effects of adjuvant radiotherapy and chemotherapy are relatively limited. The lungs and lymph nodes are the most common sites of metastasis for this tumor. In this article, we aim to share relevant experiences and emphasize the importance of diagnosis, differential diagnosis, and treatment of vulvar epithelioid sarcoma. Due to the extreme rarity of vulvar epithelioid sarcoma, there is a lack of clinical experience in patient management, particularly when the lesion is deep and concealed, and when it is associated with local vulvar abscesses, which can easily be confused with benign vulvar tumors. This can lead to misdiagnosis and delays in treatment, resulting in rapid disease progression and poor prognosis. Based on the above discussion, this further underscores the importance of comprehensive examination, accurate diagnosis, and timely treatment, serving as a warning for clinical patient management.

## Data Availability

The raw data supporting the conclusions of this article will be made available by the authors, without undue reservation.
